# Problematic Internet Use Among Adolescent Male and Female Psychiatric Inpatients: A Gender Perspective

**DOI:** 10.1007/s10578-022-01408-6

**Published:** 2022-09-07

**Authors:** Kornelius Winds, Marcel Aebi, Belinda Plattner

**Affiliations:** 1 Christian-Doppler-Clinic, University Clinics for Child and Adolescent Psychiatry, Paracelsus Medical University, Ignaz-Harrer-Straße 79, 5020 Salzburg, Austria; 2 Department of Justice and Home Affairs, Research & Development, Corrections and Rehabilitation, Canton of Zurich, Switzerland

**Keywords:** Problematic internet use, Adolescence, Temperament and character, Personality, Gender

## Abstract

Problematic internet use (PIU) is of treatment interest in adolescent clinical samples. Gender specific differences in terms of personality traits and psychopathological symptoms remain unclear. In an adolescent clinical sample (n = 104; 69 girls) PIU, psychopathology, temperament and character traits as well as emotional and behavioral problems were assessed. 62% of the sample showed subthreshold PIU and 34% full PIU (fPIU). Boys reported more gaming whereas girls social networking. Sex specific analyses revealed gender differences: Girls with fPIU scored significantly higher on internalizing/externalizing problems/behavior, novelty seeking and transcendence, and lower on persistence, self-directedness, and cooperativeness than girls without fPIU. Boys with fPIU scored significantly higher on internalizing problems and self-transcendence and lower on harm avoidance than boys without fPIU. Gender plays an important role in PIU. Gender specific differences in both application use as well as symptomatic, temperament and character traits call for a gender specific approach in prevention and treatment integration.

## Introduction

In child and adolescent psychiatry multimodal treatment combining psychotherapy, psychopharmacology and psychosocial interventions are state of the art. In implementing adequate care when working with the next generation, it is mandatory to continuously reflect new challenges facing this age group. Internet use and its aspects, with beneficial as well as harming sequelae seem to be of specific interest [1–3]. These include new forms of leisure activity (e.g. online computer gaming, online shopping, video and music streaming), alteration in interpersonal communication (brief verbal communication with visual focus in social networking platforms) as well as a widespread implementation into educational and work-related fields [4–12].

Especially adolescents in clinical treatment settings who have a more distinct psychopathology and tend to malfunctioning in several aspects of their everyday life do also use internet-based features and the question at hand is, if they would profit from treatment especially focussing on this behavioural aspect.

### Developmental Aspect of Problematic Internet Use

Negative behavioral, social and health consequences of internet use are summarized by the term problematic internet use (PIU). Findings of the PIU prevalence vary within the literature, with prevalence rates from 5.6% to 13.9% in the general population [13–18] and 4.2–28.8% in clinical samples (e.g. rehabilitation facility, inpatient clinic) [19–21]. Variation can be explained by the use of different measuring tools, regional differences, diverging study populations and gender differences [4, 19, 22–24]. Several factors are associated with the development and maintenance of PIU [25–27]: (1) neurobiology and genetics [28–33], (2) psychosocial aspects [2, 14, 34, 35], (3) psychological features [23, 36, 37], (4) psychopathology [15, 19, 38–41], (5) personality traits [42–47].

The Interaction of Person-Affect-Cognition-Execution (I-PACE) model aims to explain the development and maintenance of PIU by combining several factors such as predisposing factors, affective and cognitive reactions, control of executive or inhibitory functions, use of specific internet applications or sites and consequences of usage of choice due to decision making strategies. Such the development of PIU seems related to several predisposing and risk factors interacting as intensifier in a continuous process from leisure activity to non-substance related behavioral addiction. Internet-related cognitive biases (such as general dysfunctional attitudes, expectancies, illusions, implicit associations) play an important role in the maintenance of PIU [26].

### Psychopathology, Personality and PIU

Research shows that adolescents with PIU both in clinical as well as in non-clinical samples show higher prevalence rates and symptom scores of psychopathologies and functional impairment, with the biggest effect sizes associated with obsessive–compulsive and anxiety-symptoms in a clinical sample [40]. Prevalence rates of PIU in children and youth psychiatric inpatients are high. Associations between PIU and characteristic patterns of psychopathology such as difficulties to establish a stable and consolidated identity, suicidality and peer victimization have been described [19]. In terms of personality, in a recent metanalysis of studies in adult samples, five traits have been shown to correlate with PIU: Agreeableness, openness to experiences, extraversion and conscientiousness show negative, whereas neuroticism shows a positive correlation with PIU [43]. Relations between PIU and high neuroticism in a clinical adolescent sample could be found.

### Gender and Internet Use

Gender differences in the use of internet applications can be observed: Females are more engaged in searching information, watching videos and communicating via platforms and social networking sites (SNS) [4, 22, 48, 49]. Female youth show a greater chance for the development of more specific forms of PIU such as problematic social media use [50]. The personality features of female youth with PIU seem to be complex with on the one hand high scores on social as well as emotional impairment and on the other hand extraversion and positive affect [40, 49]. In terms of psychopathology conduct problems and hyperactivity/inattention was more prevalent in female youth showing PIU when compared to the males [39].

Males tend to download files, gamble, surf indiscriminately, use blogs and internet bulletin boards, use pornographic websites, and play online games [4, 22, 48, 49]. Male youth more often fulfill criteria for full PIU compared to females, but also showed a likeliness for spontaneous remission over time [50, 51]. Concerning psychopathological symptoms, males showed a stronger correlation between PIU and depressive symptoms, anxiety, and peer relationship problems than females [39].

### Study Aims

The aims of the current study therefore was to assess the prevalence of PIU in an impatient setting, to characterize and describe their personality and psychopathological profile and to focus on the gender perspective in terms of special treatment needs.

Therefore, the present study aims to test the following hypotheses:

1. The prevalence of PIU in our clinical sample will be higher than prevalence rates in the general population. Use of application forms will differ by gender.
2. There are significant differences among youth undergoing inpatient treatment with and without PIU in their prevalence of psychiatric disorder categories, self-reported emotional and behavioral problems as well as temperament and character traits.
3. There are significant gender differences among inpatient undergoing treatment with and without PIU in their prevalence of psychiatric disorder categories, self-reported emotional and behavioral problems as well as temperament and character traits.

## Methods

### Participants and Procedure

We collected data from patients treated at the Department of Child and Adolescent Psychiatry, Paracelsus Medical University between August 2018 and March 2020.

A total of 104 patients participated. All subjects as well as their custodians provided informed consent. As part of our diagnostic battery, patients completed the CIUS, the structured clinical interview of the AICA, the MINI-KID, the Youth Self-Report (YSR) and the Junior Temperament and Character Inventory (JTCI). The AICA as well as the CIUS were used to assess PIU, the Mini-International Neuropsychiatric Interview for Children and Adolescents (MINI-KID) to assess psychopathologies/comorbidities. General information on demographics such as age, gender and foreign nationality was obtained from patient files.

Inclusion criteria were the following: (1) minimum age of 14, (2) maximum age of 17, (3) undergoing inpatient treatment at the Department of Child and Adolescent Psychiatry, Paracelsus Medical University, and (4) written declaration of consent.

Exclusion criteria were the following: (1) a poor aptitude as well as (2) as well as lack of sufficient German language skills, to ensure correct completion of the self-report questionnaires, and (3) involuntary commitment. Furthermore, severe psychiatric disorders with thought disorder and loss of reality (such as psychotic disorders) present at the time of the study, which made the completion of the diagnostic interviews and questionnaires not accomplishable, were excluded. Exclusion criteria were defined based on the clinical judgement of the medical treatment team of our clinic.

### Measures

#### Compulsive Internet Use Scale (CIUS)

The CIUS was developed to measure internet use and its compulsiveness [52]. It contains of 14 items measured on a 5-point Likert scale and is based on the DSM-4 criteria for dependence-, obsessive–compulsive disorder and behavioral addictions. The items measure 5 criteria for PIU: loss of control, preoccupation (mental and behavioral), conflict (intrapersonal and interpersonal), withdrawal symptoms and coping or mood modification. It is a self-report questionnaire, which showed good validity, good reliability (Cronbach α = 0.89–0.90) and good internal consistency (Cronbach α = 0.87) [52]. The questionnaire measures internet use on a sum score ranging from 0 to 56 points. A cut-off score of 28 points confirms PIU [52]. A subthreshold PIU is identified by a cut-off of 21 points [53, 54]. PIU and subthreshold PIU in our study were coded according to the given cut off points.

#### Scale for the Assessment of Internet and Computer Game Addiction (AICA)

The AICA was developed to assess internet use [55, 56]. The structured clinical interview version is based on the Diagnostic and Statistical Manual of Mental Disorders (DSM) version 5 criteria for internet gaming disorder and the structural clinical interview for DSM. The interview provides the ability to assess the individual internet use (non-problematic, mild, moderate and severe PIU) and different subtypes of PIU. When compared to external ratings of psychotherapists the scores of the self-report version of the AICA, obtained a good diagnostic accuracy (sensitivity = 80.5%; specificity = 82.4%) as well as sound psychometric properties [15, 57]. Individuals scoring below 13 were scored with no PIU, between 13 and below 19 with subthreshold PIU and with 19 and above with full PIU.

#### Mini-International Neuropsychiatric Interview for Children and Adolescents (MINI-KID)

The MINI-KID is a structured clinical diagnostic interview for children and youths evaluating psychiatric disorder based on the DSM version 4 and the 10th revision of the International Statistical Classification of Diseases and Related Health Problems (ICD-10) [58]. It was used to evaluate ICD-10 current psychiatric disorder categories such as (1) affective disorders (e.g., depression, mania, bipolar disorders), (2) anxiety disorders (e.g., social anxiety, specific phobia, separation anxiety disorder, (3) substance related disorders (e.g. alcohol/drug abuse or dependencies), (4) attention disorders (e.g. attention-deficit/hyperactivity disorder; ADHD), (5) disruptive behavior disorders (oppositional defiant disorder, conduct disorder) and other disorders (e.g., tic disorder, eating disorder, adjustment disorder). The MINI-KID shows a good reliability and validity for the assessment of children and youth psychiatric disorders in shorter time compared to other instruments [59, 60].

#### Youth Self-report (YSR)

The YSR is a widely used instrument to measure self-reported behavioral and emotional problems in youth from 11 to 18 years during the past 6 months [61]. The YSR consists of 118 items that can be scored on a three-point rating scale (0 = not true, 1 = somewhat true, and 2 = very true) and lead to a total problem scale, two broad-band scales (internalizing and externalizing problems) and eight empirically derived first order syndrome scales (social withdrawal, somatic complaints, social problems, thought problems, anxiety/depression, attention problems, aggressive behaviors and delinquent behaviors. The YSR shows a good internal consistency, validity and reliability [62].

#### Junior Temperament and Character Inventory (JTCI)

The JTCI is self-report questionnaire to evaluate personality traits in youth, ranging from 12 to 18 years of age [63]. It consists of seven personality traits: four temperament traits and three character traits. The four temperament dimensions describe and evaluate emotional coping and reactions during development across the lifespan. The three character dimensions predict differences and challenges in the individual self-concept consisting of attitudes, values and goals which form coping strategies to their environment [64]. The temperament dimensions consist of novelty seeking, harm avoidance, reward dependence and persistence. The character dimensions include self-directedness, cooperativeness and transcendence [63, 65]. The JTCI was used in various representative clinical samples [64, 66]. It shows a good reliability and validity [67].

### Statistical and Data Analysis

The PIU variables were created combining the cut off score related scales from CIUS and AICA as shown in Table 1. To analyze sex differences for demographics and CIUS/AICA measures, diagnostic problem categories of youth with full PIU, and current psychiatric disorder categories and comorbidities independent t-tests were used for interval-scaled and chi-square statistics for dichotomous variables. Chi square statistics were used to analyze current psychiatric disorders in youth with and without full PIU. In order to avoid alpha-error accumulation by multiple comparisons for psychiatric disorder categories in the total sample as well as in subsamples of girls and boys, the Benjamini–Hochberg method was used for adjusting the significance level of 0.05 [68]. To analyze the effects of self-reported emotional and behavioral problems and self-reported temperament and character traits in youth with and without full PIU, t-tests and multivariate analyses of variance (MANOVA) based on General Linear Model (GLM) procedures were performed. Analyses were conducted in SPSS 26 for Apple Macintosh. Effect sizes of significant findings in chi-square tests were calculated using Cramers V with values > 0.10 interpreted as small effect, > 0.30 interpreted as medium effects, and 0.50 interpreted as large effects [69]. Effect sizes of significant t-test findings were calculated using Cohen’s d with values > 0.20 as small effect, > 0.50 interpreted as medium effects, and 0.80 interpreted as large effects [69]. Effect sizes of significant of GLM findings were calculated using partial Eta-Squares with values > 0.01 as small effect, > 0.06 interpreted as medium effects, and > 0.14 interpreted as large effects [69]. Post hoc power analyses were calculated using G*Power 3 [70]. Based on sample size of (N = 104) and bivariate comparison sufficient statistical power was found to detect large effects in t-tests / MANOVA (power = 0.744–0.968).

**Table 1:** Demographics and descriptive findings of the CIUS and AICA

	Total sample (N = 104)	Girls (n = 69)	Boys (n = 35)	Test^a^	Effect size
Demographics					
Age	15.60 (1.16)	15.52 (1.18)	15.74 (1.12)	0.92, p = .322	–
Foreign nationality	15 (14.4%)	9 (13.0%)	6 (17.1%)	0.32, p = .574	–
CIUS					
CIUS Score (m, SD)	21.45 (10.00)	20.88 (10.00)	22.57 (10.05)	0.81, p = .419	–
CIUS Subthreshold PIU (or PIU, CIUS ≥ 21; n, %)	56 (53.8%)	36 (52.2%)	20 (57.1%)	0.23, p = .631	–
CIUS PIU (CIUS ≥ 28; n, %)	29 (27.9%)	18 (26.1%)	11 (31.4%)	0.33, p = .566	–
AICA					
AICA Score (m, SD)	14.13 (8.13)	13.12 (7.77)	16.11 (8.57)	1.80, p = .075	–
AICA mild PIU (or moderate/severe, AICA ≥ 13; n, %)	52 (50.0%)	31 (44.9%)	21 (60.0%)	2.11, p = .146	–
AICA moderate/severe PIU (AICA ≥ 19; n, %)	**29 (27.9%)**	**15 (21.7%)**	**14 (40.0%)**	**3.85, p = .050**	–
Subthreshold PIU (CIUS ≥ 21 or AICA ≥ 13, n, %)	65 (62.5%)	43 (62.3%)	22 (62.9%)	.003, p = .957	–
Full PIU (CIUS ≥ 28 or AICA ≥ 19, n, %)	36 (34.6%)	21 (30.4%)	15 (42.9%)	1.58, p = .208	–

Statistically significant results are given in bold

^a^Independent sample t-test for continuous measures and Χ2-test for categorical measures

*m* mean, *SD* standard deviation, *CIUS* Compulsive internet use scale, *PIU* Problematic internet use, *AICA* Assessment of internet and computer game addiction

### Ethics

This study was approved by the ethics committee of the state Salzburg and was performed according to the Declaration of Helsinki 1995 (as revised in Edinburgh in 2000). All participants and their legal custodians provided written informed consent prior to initiation of research.

## Results

Out of the eligible population (n = 141) 10 refused to take part in the study and 27 were excluded due to not meeting other study exclusion criteria: 6 with poor aptitude, 13 with insufficient knowledge of the German language, 8 with severe psychiatric disorders with thought disorder and/or loss of reality. Findings for descriptive and CIUS/AICA measures in the total sample and boys/girls subsamples are shown in Table 1. Based on the combined measures for assessing problematic internet use with the CIUS and the AICS more than 62% of the total sample were found to have at least subthreshold PIU and more than 34% were found to have fPIU. No significant sex differences were detected. The analyses of diagnostic problem categories of youth with fPIU show that most of them were consuming online videos, SM, SNS and online computer games (Table 2). Boys with fPIU were found to play more online computer games whereas girls were more active in SNS (Table 2).

**Table 2:** Diagnostic problem categories of youth with full PIU

AICA Diagnostic problem categories	Full PIU (n = 36) n (%)	Girls (n = 21) n (%)	Boys (n = 15) n (%)	Χ^2^-test	Effect-size^a^
Online computer games	**11 (30.6%)**	**1 (4.8%)**	**10 (66.7%)**	**p < .001**^**b**^	**.66**
Offline computer games	0 (0.0%)	0 (0.0%)	0 (0.0%)	–	–
Online Pornography	0 (0.0%)	0 (0.0%)	0 (0.0%)	–	–
Online gambling (casino)	0 (0.0%)	0 (0.0%)	0 (0.0%)	–	–
Online shopping	0 (0.0%)	0 (0.0%)	0 (0.0%)	–	–
Social media	**21 (58.3%)**	**16 (76.2%)**	**5 (33.3%)**	**6.61, p = .010**	**.43**
Social networks	**12 (33.3%)**	**11 (52.4%)**	**1 (6.7%)**	**p = .005**^**b**^	**.48**
Online information	0 (0.0%)	0 (0.0%)	0 (0.0%)	–	–
Online videos	26 (72.7%)	14 (66.7%)	12 (80.0%)	p = .468^b^	–
others	5 (13.9%)	4 (19.0%)	1 (6.7%)	p = .376^b^	–

Statistically significant results are given in bold

^a^Cramers V, interpretation according to (Cohen [69]) 0.10 (small effect), 0.30 (medium effect), and 0.50 (large effect)

^b^Fishers exact test

Psychiatric disorders and comorbidities were frequently diagnosed; 99% met the criteria of at least one psychiatric disorder according to the MINI-KID and more than 86% met criteria for at least one comorbid disorder (Table 3).

**Table 3:** Current psychiatric disorders and comorbidities (based on MINI-KID interviews)

	Total sample (N = 104) n (%)	Girls (n = 69) n (%)	Boys (n = 35) n (%)	Χ^2^-test	Effect-size^a^
Diagnostic categories					
Affective disorders	75 (72.1%)	53 (76.8%)	22 (62.9%)	2.25, p = .134	–
Anxiety disorders	**86 (82.7%)**	**62 (89.9%)**	**24 (68.6%)**	**7.35, p = .007**	**.27**
Substance use disorders	47 (45.2%)	27 (39.1%)	20 (57.1%)	3.04, p = .081	–
Attention disorders	26 (25.0%)	14 (20.3%)	12 (34.3%)	2.43, p = .119	-
Disruptive behavior disorders	45 (43.3%)	26 (37.7%)	19 (54.3%)	2.61, p = .106	–
Other disorders	25 (24.0%)	17 (24.6%)	8 (22.9%)	0.40, p = .841	–
Any disorder	103 (99.0%)	68 (98.6%)	35 (100%)	p = 1.000^b^	–
One or more comorbid categories	90 (86.5%)	62 (89,9%)	28 (80,0%)	1.94, p = .164	–
Two or more comorbid categories	58 (55.5%)	38 (55.1%)	20 (57.1%)	0.04, p = .841	–
Three or more comorbid categories	32 (30.8%)	20 (29.0%)	12 (34.4%)	0.31, p = .580	–

Statistically significant results are given in bold

^a^Cramers V, interpretation according to (Cohen [69]) 0.10 (small effect), 0.30 (medium effect), and 0.50 (large effect)

^b^Fishers exact test, statistically significant results are bold

Prevalence rates of psychiatric diagnostic categories and any psychiatric disorder did not differ for youth with and without fPIU (Table 4). Sex specific analyses revealed that girls with fPIU more often showed disruptive behavior disorders than girls without fPIU. No further differences were found for further psychiatric diagnostic categories in the girl and in the boy subsample.

**Table 4:** Current psychiatric disorders (based on MINI-KID interviews) in youth with and without full PIU

	Total sample (N = 104)			Girls (n = 69)				Boys (n = 35)				
	Full PIU 36 34.6(%) n (%)	No full PIU 68 (65.4%) n (%)	Χ^2^-test^1^	Effect-size^a^	Full PIU 21 30.4(%) n (%)	No full PIU 48 (69.6%) n (%)	Χ^2^-test^1^	Effect- size^1^	Full PIU 15 (57.1%) n (%)	No full PIU 20 (42.9%) n (%)	Χ^2^-test^1^	Effect-size^a^
Affective disorders	29 (80.6)	46 (67.6)	1.95, p = .571	–	18 (85,7)	35 (72.9)	1.34, p = .574	–	11 (73.3)	11 (55.0)	1.23, p = 1.000	–
Anxiety disorders	30 (34.9)	56 (56.1)	0.02, p = .900	–	19 (90.5)	43 (89.7)	0.01, p = .910	–	11 (73.3)	13 (65.0)	0.28, p = .699	–
SUD	19 (52.8)	28 (41.2)	1.28, p = .602	–	12 (57.1%	15 (31.3)	4,11, p = .151	–	7 (46.7)	13 (65.0)	1.17, p = .970	–
Attention disorders	10 (27.8)	16 (23.5)	0.23, p = .739	–	6 (28.6)	8 (16.7)	1.28, p = .452	–	4 (26.7)	8 (40.0)	p = .685^b^	–
DBD	20 (55.6)	25 (36.8)	3.39, p = .462	–	**13 (61.9)**	**13 (27.1)**	**7.54, p = .042**	**.33**	7 (46.7)	12 (60.0)	0.61, p = .758	–
Other disorders	10 (27.8)	15 (22.1)	0.42, p = .722	–	6 (28.6)	11 (22.9)	0.25, p = .719	–	4 (26.7)	4 (20.0)	p = .700^b^	–
Any disorders	34 (94.4)	67 (98.5)	p = .480^b^	–	20 (95.2)	47 (97.9)	p = .727^b^	–	14 (93.9)	20 (100.0)	p = 1.000^b^	–

Statistically significant results are given in bold

^1^corrected for multiple testing according to Benjamini Hochberg method (Benjamini and Hochberg [68])

^a^Cramers V, interpretation according to (Cohen [69]) 0 .10 (small effect), 0.30 (medium effect), and 0.50 (large effect)

^b^Fisher’s Exact Test

*PIU* Problematic internet use, *SUD* Substance use disorders, *DBD* disruptive behavior disorders

The multivariate analyses for self-reported emotional and behavioral problems yielded a number of significant differences between youth with and without fPIU (Table 5). Youth with fPIU were scoring higher on the YSR total score as well as on the YSR internalizing and externalizing broad band scales. Furthermore, youth with fPIU scored higher on all YSR subscales except YSR thought problems. Additional sex specific analyses revealed that girls with fPIU scored higher on the YSR total score, YSR internalizing and externalizing problems as well as on YSR anxious depressed, YSR social problems, YSR attention problems, YSR delinquent behavior and YSR aggressive behavior subscales than girls without fPIU. In contrast, boys with fPIU scored higher on the YSR total score and YSR internalizing (but not on YSR externalizing) as well as on YSR somatic complaints, YSR anxious depressed, YSR social problems subscales than boys without fPIU.

**Table 5:** Self-reported emotional and behavioral problems and self-reported temperament and character traits in youth with and without full PIU

	Total sample (N = 104)				Girls (n = 69)				Boys (n = 35)			
	Full PIU m (SD)	No full PIU m (SD)	Test^a^	Effect-size	Full PIU m (SD)	No full PIU m (SD)	Test^a^	Effect-size	Full PIU m (SD)	No full PIU m (SD)	Test^a^	Effect-size
YSR total score	**83.97 (27.83)**	**61.29 (23.77)**	**4.36, p < .001**	**0.88**^**b**^	**87.67 (30.21)**	**63.69 (23.29)**	**3.59, p = .001**	**0.89**^**b**^	**78.80 (24.15)**	**55.55 (24.54)**	**2.79, p = .009**	**0.96**^**b**^
**YSR broad-band scales**												
YSR externalizing	**21.25 (9.82)**	**13.69 (8.94)**	**15.72, p < .001**	**.134**^**c**^	**22.95 (10.32)**	**12.71 (8.88)**	**17.60, p < .001**	**.208**^**c**^	18.87 (8.86)	16.05 (8.86)	.087, p = .359	–
YSR internalizing	**32.56 (9.55)**	**24.99 (11.44)**	**11.50, p = .001**	**.101**^**c**^	**34.14 (10.40)**	**27.17 (10.73)**	**6.29, p = .015**	**.086**^**c**^	**30.33 (8.03)**	**19.75 (11.65)**	**9.10, p = .005**	.**216**^**c**^
**YSR syndrome scales**												
YSR social withdrawal	**8.06 (2.87)**	**6.54 (3.47)**	**5.02, p = .023**	**.047**^**c**^	8.29 (3.04)	6.71 (3.38)	3.38, p = .070	–	7.73 (2.69)	6.15 (3.73)	1.94, p = .173	–
YSR somatic complaints	**5.89 (3.03)**	**4.47 (3.36)**	**4.48 p = .037**	**.042**^**c**^	6.05 (3.10)	5.23 (3.47)	0.87 p = .356	–	**5.67 (3.02)**	**2.65 (2.28)**	**11.39 p = .002**	**.257**^**c**^
YSR anxious/depressed	**20.19 (6.48)**	**15.26 (7.52)**	**11.08, p = .001**	**.098**^**c**^	**21.38 (7.07)**	**16.54 (6.95)**	**7.02, p = .010**	**.095**^**c**^	**18.53 (5.36)**	**12.20 (8.14)**	**6.83, p = .013**	**.171**^**c**^
YSR social problems	**5.42 (3.10)**	**3.25 (2.40)**	**16.57 p < .001**	**.132**^**c**^	**5.22 (3.22)**	**3.50 (2.48)**	**6.62 p = .012**	**.090**^**c**^	**5.53 (3.04)**	**2.65 (2.13)**	**10.87 p = .002**	**.248**^**c**^
YSR thought problems	3.75 (3.26)	3.32 (2.96)	0.45, p = .501	–	3.57 (3.12)	3.73 (3.09)	0.04, p = .846	–	4.00 (3.55)	2.35 (2.43)	2.67, p = .112	–
YSR attention problems	**9.47 (3.52)**	**7.00 (3.32)**	**12.52, p = .001**	**.109**^**c**^	**9.71 (3.77)**	**6.83 (3.52)**	**10.54, p = .002**	**.136**^**c**^	9.13 (3.23)	7.40 (3.60)	2.17, p = .151	–
YSR delinquent behaviors	**7.58 (3.95)**	**5.15 (3.67)**	**9.86, p = .002**	**.088**^**c**^	**8.57 (3.98)**	**4.54 (3.67)**	**16.71 p < .001**	**.200**^**c**^	6.20 (3.57)	6.60 (3.30)	0.12 p = .734	–
YSR aggressive behaviors	**13.67 (6.79)**	**8.54 (5.96)**	**15.78, p < .001**	**.134**^**c**^	**14.38 (7.23)**	**8.17 (5.70)**	**14.70 p < .001**	**.180**^**c**^	12.69 (6.22)	9.45 (6.61)	2.14 p = .153	–
**JTCI Temperament**												
JTCI novelty seeking	**33.02 (11.12)**	**27.94 (11.99)**	**4.45, p = .037**	**.042**^**c**^	**32.14 (10.89)**	**25.62 (11.25)**	**5.02 p = .028**	**.070**^**c**^	34.27 (11.80)	33.50 (12.16)	0.04, p = .853	–
JTCI harm avoidance	**34.98 (7.59)**	**30.04 (9.45)**	**4.90, p = .029**	**.046**^**c**^	35.00 (7.27)	32.48 (8.18)	1.48, p = .228	–	**32.80 (8.09)**	**24.20 (9.91)**	**7.52, p = .010**	**.185**^**c**^
JTCI reward dependence	40.04 (8.90)	36.75 (12.37)	1.98, p = .162	-	39.19 (8.47)	37.17 (12.56)	0.45, p = .503	–	41.20 (9.65)	35.75 (12.17)	2.04, p = .163	–
JTCI persistence	**25.58 (9.55)**	**30.99 (11.02)**	**6.18, p = .015**	**.057**^**c**^	**22.39 (8.30)**	**33.17 (9.86)**	**19.15, p < .001**	**.222**^**c**^	30.07 (9.64)	25.75 (12.12)	1.29, p = .265	–
**JTCI Character**												
JTCI self-directedness	**22.42 (11.09)**	**27.75 (10.18)**	**6.07, p = .015**	**.056**^**c**^	**20.81 (10.24)**	**26.92 (9.67)**	**5.62, p = .021**	**.077**^**c**^	24.67 (12.19)	29.75 (11.31)	1.62, p = .212	–
JTCI cooperativeness	10.44 (3.18)	11.54 (3.13)	2.87, p = .093	–	**10.05 (3.26)**	**11.94 (3.24)**	**4.95, p = .029**	**.069**^**c**^	11.00 (3.09)	10.60 (2.70)	0.17, p = .686	–
JTCI transcendence	**19.97 (7.68)**	**13.94 (7.18)**	**15.83, p < .001**	**.134**^**c**^	**19.57 (7.55)**	**14.06 (7.77)**	**7.73, p = .007**	**.103**^**c**^	**20.40 (8.11)**	**13.65 (5.67)**	**8.42, p = .007**	**.203**^**c**^

Statistically significant results are given in bold

^a^t-test for YSR total score, General Linear Model (GLM) based on YSR broad-band scales (total sample): Wilks Lambda, F = 10.29, df = 2, p < .001, GLM based on YSR problem syndrome scales (total sample): Wilks Lambda, F = 3.47, df = 8, p = .001, GLM based on JTCI temperament scales (total sample): Wilks Lambda, F = 4.46, df = 4, p = .002, GLM based on JTCI character scales (total sample): Wilks Lambda, F = 6.63, df = 3, p < .001, GLM based on YSR broad-band scales (girls): Wilks Lambda, F = 9.34, df = 2, p < .001, GLM based on YSR problem syndrome scales (girls): Wilks Lambda, F = 3.34, df = 8, p = .003, GLM based on JTCI temperament scales (girls): Wilks Lambda, F = 5.67, df = 4, p = .001, GLM based on JTCI character scales (girls): Wilks Lambda, F = 4.65, df = 3, p < .005, YSR GLM based on YSR broad-band scales (boys): Wilks Lambda, F = 4.46, df = 2, p = .020, GLM based on YSR problem syndrome scales (boys): Wilks Lambda, F = 2.90, df = 8, p = .019, GLM based on JTCI temperament scales (boys): Wilks Lambda, F = 3.36, df = 4, p = .022, GLM based on JTCI character scales (boys): Wilks Lambda, F = 3.47, df = 3, p < .028

^b^Cohen’s d, interpretation according to Cohen [69] 0.20 (small effect), 0.50 (medium effect), and 0.80 (large effect)

^c^Partial Eta-Square interpretation according to Cohen [69] 0 .01 (small effect), 0.06 (medium effect), and 0.14 (large effect)

*YSR* Youth self report, *JTCI* Junior temperament and character inventory

Finally, self-reported temperament and character traits were found to differ in youth with and without fPIU (Table 5). Youth with fPIU scored higher on JTCI novelty seeking and JTCI transcendence but lower on JTCI harm avoidance, JTCI persistence, and JTCI self-directedness than youth without fPIU. Temperament and character traits were found more often to differ between girls with and without fPIU than in boys with and with fPIU: Girls with fPIU scored higher on JTCI novelty seeking and JTCI transcendence but lower on JTCI persistence, JTCI self-directedness, and JTCI cooperativeness than girls without fPIU. Boys with fPIU only scored higher on JTCI transcendence and lower on JTCI harm avoidance than boys without full PIU.

## Discussion

To expand previous research on PIU, the current study examined the prevalence of PIU and specific usage behavior in both genders as well as differences in the prevalence of psychiatric disorder categories, self-reported emotional and behavioral problems as well as temperament and character traits between youths undergoing inpatient treatment with and without PIU. Over 30% of our inpatient sample showed PIU (based on fPIU criteria according to CIUS/AICA). Gender differences could be observed in the use of individual internet applications, with higher numbers of problematic computer gaming in boys and higher numbers of problematic SM and SNS use in girls. Diagnostic categories were not useful to discriminate PIU and non-PIU in our inpatient sample. However, a symptom and personality approach showed significant differences between those with PIU and those without it. The PIU prevalence rates in our inpatient sample were higher than those in the general population, confirming previous results [48] as well as those reported in recent studies in clinical youth [19]. This could be explained by the additional use of a diagnostic interview rather than a self-report alone.

The high levels of PIU in adolescents in a psychiatric treatment setting could be understood as a form of escapism. PIU might function as a strategy to cope with emotional difficulties and stressful life events [71]. Depression, anxiety, introversion, negative affect and emotional instability may facilitate pathways for reduced behavioral self-limitation and the development of dysfunctional behaviors such as the overuse of the internet [71–75].

Interestingly, there were no gender differences in the prevalence of PIU observed in our sample. Yet, the use of internet application differs significantly between boys and girls, with boys showing problematic computer gaming, and girls showing problematic use of SM and SNS. These findings where consistent with findings from community samples [4, 22, 50]. Such, addressing PIU in treatment settings should warrant a gender specific approach, raising the question if psychopathology originates from or is at least involved in the development of PIU.

Psychiatric disorder categories did not discriminate youth with or without PIU, with exception of girls in which disruptive behavior was associated with PIU. In previous studies, ADHD has been shown to be prevalent among adolescents referred for PIU [38]. Oppositional defiant disorder and conduct disorder are often linked to ADHD as being the most likely comorbid condition [76] and were also found in adolescents referred for PIU [38].

In contrast to clinical categories, a symptom and personality approach was more suitable to characterize PIU in a clinical sample. In girls PIU was significantly associated with externalizing as well as internalizing symptoms, anxious/depressed traits, social problems, attention problems, delinquent behavior, aggressive behavior, high novelty seeking, low persistence, low self-directedness, low cooperativeness and high transcendence.

Boys showed significant associations between PIU and internalizing symptoms, somatic complaints, anxious/depressed traits, social problems, low harm avoidance as well as high transcendence.

Gender specific differences might develop due to different patterns of application use in boys and girls. As shown in previous studies and confirmed by our finding girls tend to use the internet rather for social interaction and attention seeking [4, 77–79]. Problematic SNS use has been associated with higher levels of neuroticism and impulsivity [80, 81]. Extraverted users tend to present themselves with higher frequency of posting activity, larger online networks and higher counts of “likes”. Neurotic users show higher numbers of posted words or comments resulting in an increased posting activity as well as lower numbers of “likes” [82, 83].

Previous studies show an association between problematic use of various digital technologies and the fear of missing out (FoMO) [84, 85]. FoMO is the tendency to develop anxiety when missing out on rewarding experiences of others leading to the need of staying constantly connected to the social network [86]. Previous studies show that FoMO is being more related to the female gender [87, 88]. Furthermore, negative consequences such as cybervictimization and problematic communication patterns might develop over time. Studies show that girls are more likely to become cyber-victims [89, 90]. Cyberbullying and cybervictimization were associated with suicidal thoughts and suicide attempts [91].

We found significant correlations in both gender groups for self-transcendence. Self-transcendence is a character trait implying identification with everything envisioned as essential as well as being a part of a spiritual union [63]. High levels of self-transcendence were found to contribute to the severity of gambling behavior [92]. Irrational attitudes appear within this personality trait and play a role in maintaining gambling behavior [93]. We assume that high levels of self-transcendence bear the risk of losing reality and can cause difficulties in maintaining daily routine as well as sustaining problematic internet use.

The strength of our study was the comprehensive test battery, with a categorical and symptomatic approach for the assessment of psychopathology and personality assessment. To detect PIU a combination of a self-report and an interview were used [52, 55]. This allowed us to combine the findings of two valid diagnostic instruments and assured the diagnostic suitability in our sample. Another strength of our study was, that patients were interviewed by one child and adolescent psychiatrist.

Some study limitations are worth noting. First, our sample showed a ceiling effect for psychopathology. Second, although the prevalence of fPIU in our sample was higher than in general adolescent populations, given the relatively low prevalence of PIU in general, the resulting fPIU sample size is rather small. Aware of these facts effect sizes are reported for the statistical analyses. Third, our sample was imbalanced with more females. Finally, in order to minimize potential effects of treatment on the incidence of PIU, the assessment took part shortly after hospital admission; nevertheless the effects of treatment can not be fully excluded. In substance related addiction psychopharmacology has been shown effective in treatment [94]. In non-substance related addictions, the benefit of medication is not yet clear. In future research more emphasis should be given on the influence of medication on the development of PIU.

## Summary

Dealing with “the next generation” child and adolescent psychiatry has to keep on track with social developments and consider the new challenges that children and adolescents face in the implementation of diagnostic and therapeutic approaches. High prevalence rates of PIU in child and adolescent psychiatric populations underline the need for a more profound understanding of psychopathological and personality mechanisms associated with PIU: Are specific psychopathologies or personality traits associated with PIU? Which form of internet applications are used and most importantly is there a gender difference in usage of new media and do associated pathologies and personality aspects differ within gender? Therefore, the study was conducted to investigate gender differences and their associations with the prevalence of PIU, comorbidities of PIU as well as psychological and personality traits in an adolescent psychiatric population. Our results show that more than half of our sample presented with subthreshold PIU (62%) and more than a quarter reported (34%) fPIU. Girls used SNS more often and in girls the pathological use was associated with externalizing behavior such as disruptive behavior disorder, novelty seeking, low persistence, self-directedness, and cooperativeness. Boys with fPIU scored significantly higher on internalizing problems and lower on harm avoidance than boys without fPIU. Addressing PIU is unavoidable in child and adolescent psychiatric settings. The patterns of internet behavior show gender specific differences. Girls with externalizing behavior are at risk for overinvolvement in social media and boys with internalizing behavior for withdrawal into computer gaming. There is a need for psychoeducational and treatment approaches targeting PIU in child and adolescent inpatient settings.
